# Amodal completion and relationalism

**DOI:** 10.1007/s11098-022-01777-7

**Published:** 2022-04-28

**Authors:** Bence Nanay

**Affiliations:** 1 Centre for Philosophical Psychology, University of Antwerp, D 413, Grote, Kauwenberg 18, 2000 Antwerp, Belgium; 2 Peterhouse, University of Cambridge, Cambridge, CB2 1RD UK

**Keywords:** Amodal completion, Perceptual representation, Representationalism, Relationalism, Perceptual phenomenology

## Abstract

Amodal completion is usually characterized as the representation of those parts of the perceived object that we get no sensory stimulation from. In the case of the visual sense modality, for example, amodal completion is the representation of occluded parts of objects we see. I argue that relationalism about perception, the view that perceptual experience is constituted by the relation to the perceived object, cannot give a coherent account of amodal completion. The relationalist has two options: construe the perceptual relation as the relation to the entire perceived object or as the relation to the unoccluded parts of the perceived object. I argue that neither of these options are viable.

## Amodal completion

Amodal completion is usually characterized as the representation of those parts of the perceived object that we get no sensory stimulation from. The most well-known example of amodal completion is in the visual sense modality, where it comprises the representation of occluded parts of objects we see: when we see a cat behind a picket fence, our perceptual system represents those parts of the cat that are occluded by the picket fence.

Amodal completion also happens in other sense modalities. In the auditory sense modality, for example, when we hear a loud bang while listening to a tune, the auditory system continues to represent the tune even in that brief moment when the bang is the only auditory stimulation. What we have here is a form of temporal occlusion, where the bang ‘occludes’ part of the tune. Amodal completion also happens in the olfactory sense modality (Young and Nanay in press).

Amodal completion is the norm, not the exception. We are very rarely in a perceptual scenario where there is no amodal completion: in natural scenes we always get occlusion because objects tend not to be fully transparent. Given that amodal completion is an important part of the vast majority of our perceptual states, no theory of perception can be considered complete if it can’t account for this phenomenon.

We now understand the mechanism of amodal completion fairly well. In humans and nonhuman primates, the main visual pathway connects neural networks in the retina to the primary visual cortex (V1) via the lateral geniculate nucleus (LGN) in the thalamus; outputs from V1 activate other parts of the visual cortex and are also fed forward to a range of extrastriate areas (V2, V3, V4/V8, V3a, V5/MT). The primary visual cortex (and also many other parts of the visual cortex; see Grill-Spector and Malach 2004 for a summary) is organized in a way that is structurally homomorphic to the retina–it is retinotopic. If you are looking at a triangle, there is a triangle-pattern activation of direction-sensitive neurons in your primary visual cortex.

**Fig. 1:**
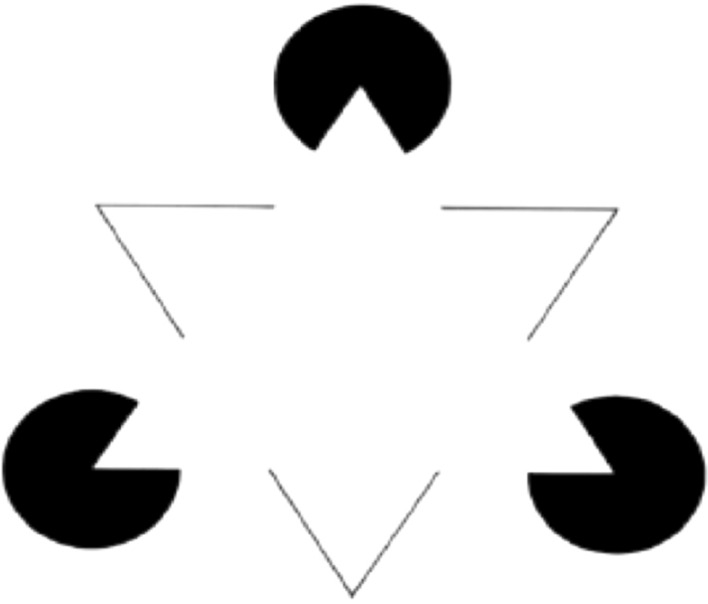
The Kanizsa triangle

But what happens if the triangle you are looking at is partly occluded? In this case, on the retina some parts of the triangle are missing, but these missing parts show up in the primary visual cortex (see Lee & Nguyen, 2001; Komatsu, 2006; Scherzer & Ekroll, 2015; Vrins et al., 2009; Lommertzen et al., 2009; Smith & Muckli, 2010; Bakin et al., 2000; Ban et al., 2013; Bushnell et al., 2011; Emmanuoil & Ro 2014; Hazenberg et al., 2014; Lee et al., 2012; Pan et al., 2012; Shibata et al., 2011; Hedge’ et al., 2008; Kovacs et al., 1995; Sugita, 1999). This is what happens when we look at the Kanizsa triangle (Fig. 1)^1^.

Amodal completion is not a perceptual curiosity: it is part of our ordinary perception. It happens very rarely in real life situations that we can perceive an object without exercising amodal completion: in natural scenes we always get occlusion because objects tend not to be fully transparent. Every time we see an object occluded by another object (which means almost every time we see anything in real life, barring odd cases of fully transparent visual scenes or very simple visual displays), we use amodal completion of the occluded parts of perceived objects (Bakin et al., 2000).^2^

For the purposes of this paper, I would like to set aside the ongoing debate about just what kind of mental states are responsible for amodal completion: the debate about whether amodal completion is a form of perception, a form of mental imagery or maybe belief (Briscoe, 2011; Nanay, 2010a, 2010b)—although I will say a bit more about it in Sect. 4. below.

Amodal completion may be, but it does not have to be, sensitive to top-down influences (Hazenberg & Van Lier, 2016; Hazenberg et al., 2014). Some instances of amodal completion may be fully bottom-up driven, like the completion of shapes purely on the basis of Gestalt forms (that can go against our best judgments). But some other times, amodal completion is driven in a top-down manner as in the case of seeing the cat behind the picket fence. Depending on what cats I encountered before, the way I complete this figure would be very different. The same goes for the amodal completion of letters and words. Higher order knowledge and expectations play an important role here.

Further, amodal completion may be conscious or unconscious. Given the sheer amount of amodal completion the visual system needs to do at any given moment, amodal completion is normally unconscious. When I see fifty cats behind the picket fence, I do not form a conscious representation of all occluded parts of all the fifty cats. But amodal completion can be conscious if, for example, we are really interested in some of the occluded features. If for some reason I need to attend to the left eye of one of these fifty cats and it is occluded by the fence, I am likely to represent this left eye consciously.

The aim of this paper is to argue that relationalism about perception, the view that perceptual experience is constituted by the relation to the perceived object, cannot give a coherent account of amodal completion. The relationalist has two options: construe the perceptual relation as the relation to the entire perceived object or as the relation to the unoccluded parts of the perceived object. I argue that neither of these options are viable. After outlining the major tenets of relationalism in Sect. 2, I consider these two ways in which relationalists can explain amodal completion in Sections 4 and 5 respectively.

## Relationalism

In the last section, I characterized amodal completion as the representation of those parts of the perceived object that we get no sensory stimulation from. Assuming that amodal completion is a perceptual phenomenon (more about this assumption below), this definition itself takes it for granted that perceptual states represent their objects. But not everybody would be happy to take this for granted.

Representationalists say that perceptual states are representations: they represent individuals as having properties (see Siegel, 2010a, 2010b; Pautz, 2010; Tye, 2007; Crane, 2006; Burge, 2005; Peacocke, 1989; Schellenberg, 2010 for very different versions of representationalism). When I look out of the window, I see dark clouds. I perceptually represent the clouds as having the property of being dark. Things may go wrong, of course; I may have an eye condition that makes me see dark clouds, whereas the clouds are in fact very light. In this case, my perceptual state misrepresents. If I see dark clouds and the clouds are in fact dark, my perceptual state represents correctly.

Not all philosophers of perception are representationalists. Some are relationalists (or ‘naïve realists’)^3^: they claim that perceptual states are not representations (or, sometimes more modestly, that the phenomenal character of perceptual stares is not explained by representations, see Campbell, 2002; Martin, 2004, 2006; Travis, 2004; Brewer, 2006, 2011; Fish, 2009; Logue, 2012; Crowther, 2010, French 2019 for very different versions of relationalism). Perceptual states do not represent the perceived object. Rather, they have the perceived object as one of their actual constitutive parts. Or, to put it differently, relationalists claim that perceptual states are relations between the subject and the perceived object (and maybe some third relatum labeled as ‘the third relatum’ or ‘the standpoint’ (Brewer, 2011; Campbell, 2002)). So the perceived object is not something that may or may not be present when you perceive (as some representationalists would say). It has to be there for your perceptual state to be a perceptual state.

Not all relationalists deny any role representations can play in perception. A famous early characterization of the position—“the actual objects of perception… partly constitute one’s conscious experience” (Martin, 1997, p. 93)—deliberately leaves open the possibility that representations may also partly constitute one’s conscious experience. However, much of the relationalism literature is motivated by some form of skepticism about the very idea of perceptual representations (see esp. Travis, 2004; Brewer, 2006, 2011; Fish, 2009). I will set those versions of relationalism that push for some kind of mixed relationalist/representationalist accounts (e.g., Logue, 2012, see also Brewer, 2006 and McDowell, 1994) aside for the purposes of this paper.

One implication of relationalism is that hallucinations are, at least on one straightforward way of understanding hallucinations (see Byrne & Logue, 2008 for a nuanced analysis), not perceptual states: their object is missing—so they cannot be a constitutive part of the perceptual state. Many relationalists are happy to bite this bullet: hallucinations may feel like perceptual states, but they are not—they are in fact radically different: perceptual states are relations to something actual, whereas hallucinations are something different–whatever hallucinations are, they are by definition not relations to something actual.

Relationalism is very explicitly an account of the phenomenology of perception (with the possible exception of Travis, 2004). Many of the motivations for this view allude explicitly to phenomenology, for example (see, e.g., Martin, 2002, 2004, 2006; Brewer, 2011). And this is something most proponents of relationalism would be very happy to acknowledge. So all the claims about perceptual states I attributed to the relationalist are really claims about conscious perceptual experiences (there is less unity in the representationalist camp, where some take representationalism to be a view about conscious perceptual experiences, while others take it to be a view about perceptual states in general (conscious or unconscious)).

In this sense, relationalism could be consistent with the view that perceptual states are representations, as long as the perceptual phenomenology of conscious perceptual experiences is not explained by the representational properties of these perceptual experiences, but rather by the perceptual relation between the subject and the perceived object.

## The dilemma

Relationalism has been criticized from various angles. It has been argued that it cannot account for unconscious perception or else it is forced to take conscious and unconscious perception to be radically different phenomena (Berger and Nanay, 2016, see also Anaya & Clarke, 2017; Phillips, 2018a, 2018b). Or that it cannot explain the top-down influences on perception (Campbell & Cassam, 2014, see also Siegel, 2011, MacPherson, 2012, Teufel & Nanay, 2017). Or the attentional modulation of perception (see Brewer, 2013, 2017; Campbell & Cassam, 2014 for discussion). Or the crossmodal binding of different sense modalities (Nanay 2014).

My aim is to give a new argument against relationalism, namely, that it can’t give a coherent account of amodal completion (and I will focus on the visual sense modality for simplicity). The main dilemma the relationalist faces is this. Given that they take perceptual states to be partly constituted by the perceived object, they would need to specify what this perceived object would be in the case of amodal completion. It could be the entire object (some parts of which are occluded). Or it could be the unoccluded part of the object. I will argue that neither horn of this dilemma is viable. I will consider the unoccluded parts view first (in Sect. 4) and then turn to the entire object view (in Sect. 5).

## The unoccluded parts view

The first option for the Relationalist is to say that in the case of amodal completion, the perceptual relation is a relation to the unoccluded parts of the perceived object only.^4^ In the familiar case of seeing the cat behind the picket fence, my perceptual state is constituted by the relation to the little cat-slices visible through the slats.

The question then is how we can explain our experience of the occluded parts of the cat. The relationalist can’t take this experience to be a perceptual experience given that perceptual experience, according to the relationalist, is constituted by the relation to the perceived object and the occluded parts of the cat are, by the starting supposition of this horn of the dilemma, not part of either relata of this relation.

They could say that the experience is a non-perceptual one, maybe the experience of having some form of belief about the occluded parts of the perceived object. Here is a serious empirical problem with this proposal: We have plenty of evidence that amodal completion happens very early in perceptual processing. It is well-documented that there is early cortical processing in amodal completion and even processing already in the primary visual cortex (see Lee & Nguyen, 2001; Komatsu, 2006; Smith & Muckli, 2010; Bakin et al., 2000; Ban et al., 2013; Bushnell et al., 2011; Hazenberg et al., 2014; Pan et al., 2012; Shibata et al., 2011; Hedge’ et al., 2008; Kovacs et al., 1995; Sugita, 1999).

If amodal completion happens in the primary visual cortex, it is not happening at the level of belief—it happens much earlier. But here is a possible rebuttal: maybe the early cortical activation is not amodal completion—it is a consequence of the amodal completion that is done by beliefs. So the view then would be that amodally completed properties are represented by beliefs and this, in turn, activates the primary visual cortex by means of some kind of top-down influence.

There is plenty of empirical evidence that this picture cannot be correct given what we know about the timing of amodal completion. Amodal completion in the early cortices happens within 100–200 ms of retinal stimulation (Sekuler & Palmer, 1992; Rauschenberger & Yantis, 2001—this is true even of complex visual stimuli, like faces, see Chen et al., 2009, see also Lerner et al., 2004, Yun et al., 2018 and Rauschenberger et al., 2006 for detailed studies that track the (very quick) temporal unfolding of amodal completion in different parts of the visual cortex). And this is much much shorter than the time that would be needed for perceptual processing to reach all the way up to beliefs or non-perceptual representations and then trickle all the way down again to the primary visual cortex (see Thorpe et al., 1996 and Lamme & Roelfsema, 2000 for the temporal unfolding of visual processing in non-amodal cases).

Another option open to the relationalist would be to claim that the phenomenology of the occluded parts of perceived objects is not post-perceptual or belief phenomenology, but rather the phenomenology of mental imagery. And it has indeed been suggested, within the representationalist tradition, that amodal completion represents by means of mental imagery: we have mental imagery of the occluded parts (Nanay, 2010a, b, 2018, but see Briscoe, 2011).

This, on the face of it, may sound like a possible way the relationalist could follow. If the phenomenology of amodally completed contours is not perceptual phenomenology, but the phenomenology of mental imagery, then relationalists could insist that the perceived object is the unoccluded part of the object and the occluded contours are not experienced perceptually, but in some other way, so relationalism does not have to worry about those.

For this proposal to be consistent with the empirical findings of the temporal unfolding of amodal completion just outlined, mental imagery would need to be understood (as it is routinely understood in the empirical literature) as early perceptual representation that is not triggered directly by sensory input (Kosslyn et al., 1995; Nanay, 2010a, 2016, 2017a, b, 2021a, b, c, in press; Pearson & Westbrook, 2015; Pearson et al., 2015). Given that mental imagery in this sense is really just a subspecies of perceptual representation (that is not triggered directly by sensory input), this way of thinking about amodal completion does not provide an alternative to representationalism as perceptual representations play a key role in describing the process.

In other words, the problem with this proposal is that it weakens the relationalist position to an extent that it would be difficult to keep it apart from representationalism. Assuming, as relationalists routinely do (see Martin, 2002), that mental imagery is a representational state, if perception entails amodal completion in the vast majority of perceptual scenarios, this means that the perceptual relation entails a representational state in the vast majority of perceptual scenarios. And this goes against the starting claim of relationalism, namely that perceptual experience is explained by the perceptual relation and not by any kind of perceptual representation.

To sum up, taking the perceptual relation to be a relation to the unoccluded parts of perceived objects only is not a viable option for the relationalist.

## The entire object view

The second horn of the dilemma was that the perceptual relation is a relation to the entire perceived object. Not just to the unoccluded parts, but also to the occluded ones: to the whole object (see Martin, 2017).^5^

Versions of relationalism that allow the perceptual relation to be a three-place relation between the subject, the perceived object and the ‘third relatium’ or ‘standpoint’ could also choose this horn of the dilemma. In fact, they could appeal to the concept ‘third relatium’ or ‘standpoint’ to explain why we have only “partial awareness” (Campbell, 2011) of the perceived object from a specific standpoint. If we moved our head a bit to the left, our standpoint would change, and this would thereby also change the perceptual relation (while the perceived object would remain the same).^6^

**Fig. 2:**
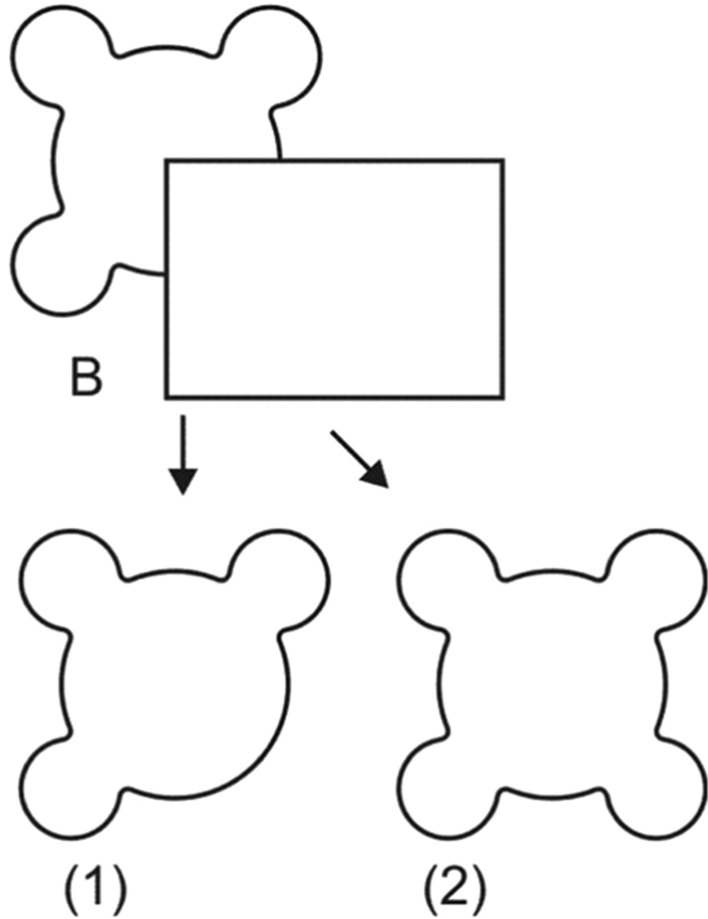
Amodal completion

The real problem, again, comes from the experience of the occluded parts of the perceived object. The experience of occluded parts of the perceived object is underspecified. Take the following example (purists may want to imagine a three-dimensional equivalent of this two-dimensional figure, where the rectangle is in fact in front):

In Fig. 2, the rectangle may occlude Shape (1) or Shape (2). Nothing in Fig. 2 tells us which of these shapes is occluded behind the rectangle. And, crucially, your experience of the occluded contour changes depending on whether you take Shape (1) or Shape (2) to be occluded behind the rectangle. You can get your experience to shift from one to the other (see Vrins et al., 2009; Hazenberg et al., 2014).

If the perceptual relation is a relation to the entire occluded object, then the question is: which of the occluded objects is it? In the case of Fig. 2, is it Shape (1) or Shape (2)? Or maybe some other shape that is indeterminate between Shape (1) and Shape (2)? Or is the question just wrongheaded because what we see is what is out there to be seen?

When seeing Shape (1) occluded in Fig. 2, we have perceptual experience P1. When seeing Shape (2) occluded in Fig. 2, we have perceptual experience P2. P1 and P2 are different and according to relationalism, this is a difference in the perceptual relation. So relationalism needs to specify what the perceptual object is.

The first thing to note is that the perceptual object can’t be the same in the two different experiences, because this would bring us back to the option that the perceptual object is the unoccluded part of the perceived object. And I hope to have dismissed that option in Sect. 4 above. So relationalists (if they take this horn of the dilemma) have to say that the perceptual object is different in these two experiences.

In other words, the relationalist who chooses the second horn of the dilemma must say that the perceptual object in P1 is Shape (1) and the perceptual object in P2 is Shape (2). But this would amount to reverting to saying that the perceptual relation is a representational relation: the perceptual object that is one of the relata of this relation is not an actual physical object out there, but it is a represented object (and that depends on how we look at Fig. 2). The same argument applies if we take the perceptual object to be some different shape, which is neither Shape (1) nor Shape (2) but rather some indeterminate shape (that maybe both of these shapes are determinates of).

Note that appealing to a ‘third relatum’ or a ‘standpoint’ won’t save the day here. The problem here is not that the perceptual object relatum of the perceptual relation underdetermines the perceptual experience. The problem here is with the perceptual object relatum of the perceptual relation alone. If the perceptual object is the same in the two experiences, we are back on the first horn of the dilemma. If it is different, then the phenomenal character of one of the two experiences remain unexplained.

For the relationalist, there is a seemingly simple answer to the question about the perceptual object in amodal completion: it is whatever actual ordinary object we are looking at. We may be, of course, fooled by the occluders into misperceiving this ordinary object. But this would be merely the case of a perceptual illusion (and relationalism has a lot to say about how to account for perceptual illusions, see, e.g., Brewer, 2011, Phillips, 2018a).

Suppose that the actual object hiding behind the rectangle is Shape (1). That is the perceptual object that constitutes one of the relata of the perceptual relation. You may of course misperceive this perceptual object. Your experience may (and is likely to) complete the figure as Shape (2). But the relationalist would explain this as an instance of perceptual illusion (maybe appealing to a form of disjunctivism). The problem with this explanatory scheme is that if the perceptual relation is between the subject and Shape (1), then the subject’s actual perceptual phenomenology as of Shape (2) remains completely unexplained. Nothing about Shape (1) (the perceptual object) and the subject’s relation to it explains this experience.

Note that the usual relationalist techniques for explaining perceptual illusions will not work. Take the best-known relationalist account of perceptual illusions, according to which what we take to be perceptual illusions are in fact post-perceptual errors (see Brewer, 2011). The general idea here is that what goes awry in what we take to be perceptual illusions has nothing to do with perception. The perceptual experience is veridical, and the non-veridicality is fully due to post-perceptual processes. While this general explanatory scheme may or may not work for illusions in general (see Phillips 2016 for some serious skepticism from the relationalist camp), it definitely won’t work to explain the phenomenology of Shape (2) because, as we have seen in Sect. 4 above, the timing of amodal completion rules out that the illusion involved here would be a post-perceptual one. Another widely discussed relationalist account of illusions posits that when we undergo perceptual illusions, something prevents us from perceiving the properties of the object we would normally perceive (Fish, 2009). In the present context it is not clear what ‘normal’ perception would mean, but even if that could be cashed out in a satisfactory manner, the phenomenal character of Shape (2) is not explained by the ‘normally perceived properties’ or whatever is preventing these from being perceived in the illusory case. Again, this is not a general worry about whether and how relationalists can deal with perceptual illusions per se (Millar, 2015), but a more specific worry about how considering the experience as of Shape (2) to be a perceptual illusion fails to explain the phenomenology.

It is important to keep apart this problem from a much more widespread potential objection to relationalism, namely, that often the same visual display will lead to very different perceptual experiences. This could be due to the differences in the allocation of attention, for example (it might also be due to differences in top-down influences on perception, but I set that case aside). Suppose that I attend to the color of the red cube in front of me. Then I shift my attention to the shape of this red cube. The visual display is the same, but the perceptual phenomenology is different.

Figure 2 could be experienced in two very different ways: as the rectangle occluding Shape (1) and as the rectangle occluding Shape (2). It is important that this is NOT the objection. When looking at Fig. 2, we could have two very different experiences of the display depending on how we complete the partly occluded shape (as Shape (1) or as Shape (2)). So our perceptual experience is not fully determined by the visual display of Fig. 2—it also depends on something that does not seem to be part of either of the two relata of the perceptual relation—neither of the perceived object nor of the subject.

But some versions of relationalism add a ‘third relatum’ (Brewer, 2011) or ‘standpoint’ (Campbell, 2002, 2011), which could, in principle explain the difference between the two experiences of seeing Shape (1) or Shape (2) occluded in Fig. 2 (if, for example, this difference could be explained in terms of differences in attention, if attention is to be subsumed under the category of the ‘third relatum’). So the relationalist can respond to the more general objection that the visual display does not determine our perceptual experience.

The more specific, more unexplored, and more serious, problem I want to raise against the relationalist is very different: According to relationalism, perceptual experience is constituted by the perceptual relation. The perceptual relation is the relation between the perceptual object and the subject (and maybe the third relatum). And even if the perceptual relation includes a third relatum, it nonetheless must include the perceptual object as one of the relata. So given that if we follow this horn of the dilemma, the perceptual object will include a necessarily representational element (something that is not in front of our eyes), this cuts off the relationalist project at its starting point.

## Conclusion

I posed a dilemma for the relationalist and argued that neither horn of the dilemma is viable. Relationalism has a real problem accounting for a crucial aspect of perception, namely, amodal completion. Given that amodal completion is not a perceptual curiosity, but rather an almost omnipresent aspect of all of our perceptual states, this is a major flaw.

## Footnotes

1 Strictly speaking, the Kanizsa triangle is an example of not amodal, but modal completion, but from a neuroscience point of view, modal and amodal completion are very similar, if not equivalent (Kellman & Shipley, 1991; Driver et al., 2001; Grossberg and Mingolla, 1985) and the differences between the two are superficial ones (Singh 2004 and Tse, 1999; Murray et al., 2004; Spehar and Halim 2016). See also the emerging research on eye movements in modal and amodal completion (Plomp et al., 2004; Desanghere and Marotta, 2015; de Grave et al., 2008; Troncoso et al., 2008).

2 There is emerging empirical evidence that the same goes for the backside of any solid object—sometimes referred to as self-occlusion. Again, we do not receive any sensory stimulation that would correspond to the backside of solid three-dimensional objects, but there is nonetheless perceptual (early cortical) processing of this missing information – in other words, amodal completion (Ekroll et al., 2016; Nanay, 2010a, 2010b). While the argument in this paper could be extended to self-occlusion, for the sake of simplicity, I will not do so here.

3 See Steenhagen (2019) on the differences between relationalism and naïve realism. Given that the argument I will present applies to both views, I will treat them as interchangeable for the purposes of this paper.

4 One prima facie consideration in favor of this horn of the dilemma is that the perceiver is in a causal relation only with the unoccluded parts of the perceived object (some relationalists would say that we are acquainted only with the unoccluded parts of the perceived object, but I will not use this term in the paper given the various senses in which it is used). There are no (direct) causal links to the occluded parts of the perceived objects. I mention this as a prima facie consideration because it would be open to a proponent of the second horn to either deny the importance of the causal link or argue that it is the entire object that is somehow in a causal relation with us.

5 One empirical reason why relationalists may want to choose this horn of the dilemma (although I don’t take this reason to be conclusive) come from recent findings that show that we treat amodally completed features as more reliable then features that are not amodally completed (Ehinger et al., 2017). If the perceptual object is the unoccluded part of the perceived object, then this poses a challenge. Given that the perceptual relation is supposed to be crucial for justifying beliefs, it would need some extra explanation if something that is not part of the perceptual object (that is, the amodally completed features) were treated as more reliable than something that is part of the perceptual object (the features that are not amodally completed). If, on the other hand, we considered the perceptual object to be the whole object (some of whose features are amodally completed), then these findings are less challenging to explain (as the amodally completed features of the perceptual object are just as relevant from a justificatory point of view as the not amodally completed ones. I said that I don’t take this to be a conclusive reason for choosing the second horn of the dilemma because of the great complexities of the question of what epistemic role amodal completion plays (see Chudnoff, 2016, 2017, Helton and Nanay, 2019).

6 **S**ee also what could be labeled as the ‘access’ view of amodal completion: Noë, 2002, 2004, 2005, the account has its roots in Pessoa et al., 1998, but see also Nanay, 2010a, 2010b for criticism, see also Leddington, 2009 for a different version of this claim).
